# Exome Sequencing of 47 Chinese Families with Cone-Rod Dystrophy: Mutations in 25 Known Causative Genes

**DOI:** 10.1371/journal.pone.0065546

**Published:** 2013-06-11

**Authors:** Li Huang, Qingyan Zhang, Shiqiang Li, Liping Guan, Xueshan Xiao, Jianguo Zhang, Xiaoyun Jia, Wenmin Sun, Zhihong Zhu, Yang Gao, Ye Yin, Panfeng Wang, Xiangming Guo, Jun Wang, Qingjiong Zhang

**Affiliations:** 1 State Key Laboratory of Ophthalmology, Zhongshan Ophthalmic Center, Sun Yat-sen University, Guangzhou, Guangdong, China; 2 BGI-Shenzhen, Shenzhen, Guangdong, China; Innsbruck Medical University, Austria

## Abstract

**Objective:**

The goal of this study was to identify mutations in 25 known causative genes in 47 unrelated Chinese families with cone-rod dystrophy (CORD).

**Methods:**

Forty-seven probands from unrelated families with CORD were recruited. Genomic DNA prepared from leukocytes was analyzed by whole exome sequencing. Variants in the 25 genes were selected and then validated by Sanger sequencing.

**Results:**

Fourteen potential pathogenic mutations, including nine novel and five known, were identified in 10 of the 47 families (21.28%). Homozygous, compound heterozygous, and hemizygous mutations were detected in three, four, or three families, respectively. The 14 mutations in the 10 families were distributed among *CNGB3* (three families), *PDE6C* (two families), *ABCA4* (one family), *RPGRIP1* (one family), *RPGR* (two families), and *CACNA1F* (one family).

**Conclusions:**

This study provides a brief view on mutation spectrum of the 25 genes in a Chinese cohort with CORD. Identification of novel mutations enriched our understanding of variations in these genes and their associated phenotypes. To our knowledge, this is the first systemic exome-sequencing analysis of all of the 25 CORD-associated genes.

## Introduction

Cone-rod dystrophy (CORD) refers to a series of hereditary retinal disorders with a predominantly cone involvement [1]. Rod impairment may occur at the same time as the cone impairment or appear later. Patients with CORD usually have reduced visual acuity, photophobia, and color vision defects.

CORD may be transmitted as an autosomal dominant (adCORD), autosomal recessive (arCORD), or X-linked trait (xlCORD). To date, mutations in at least 25 genes have been reported to be associated with different forms of CORD, including the following: aryl hydrocarbon receptor interacting protein-like 1 (*AIPL1*) [1]; the cone-rod homeobox containing gene (*CRX*) [2] *;* guanylate cyclase activator 1A (*GUCA1A*) [3] *;* guanylate cyclase 2D (*GUCY2D*) [4] *;* PITPNM family member 3 (*PITPNM3*) [5] *;* prominin 1 (*PROM1*) [6] *;* peripherin 2 (*PRPH2*) [7] *;* regulating synaptic membrane exocytosis 1 (*RIMS1*) [8] *;* sema domain, immunoglobulin domain (Ig), transmembrane domain (TM) and short cytoplasmic domain, (semaphorin) 4A (*SEMA4A*) [9] *;* unc-119 homolog (*UNC119*) [10]; ATP-binding cassette, sub-family A (ABC1) member 4 (*ABCA4*) [11] *;* ADAM metallopeptidase domain 9 (*ADAM9*) [12] *;* chromosome 8 open-reading frame 37 (*C8ORF37*) [13] *;* calcium channel voltage-dependent alpha 2/delta subunit 4 (*CACNA2D4*) [14] *;* cadherin-related family member 1 (*CDHR1*) [15] *;* ceramide kinase-like (*CERKL*) [16] *;* cyclic nucleotide gated channel beta 3 (*CNGB3*) [17] *;* cyclin M4 (*CNNM4*) [18]; potassium channel subfamily V member 2 (*KCNV2*) [19] *;* phosphodiesterase 6C, cGMP-specific, cone, alpha prime (*PDE6C*) [20] *;* retina and anterior neural fold homeobox 2 (*RAX2*) [21] *;* retinol dehydrogenase 5 (*RDH5*) [22] *;* retinitis pigmentosa GTPase regulator interacting protein 1 (*RPGRIP1*) [23]; calcium channel voltage-dependent L type alpha 1F subunit (*CACNA1F*) [24] *;* and retinitis pigmentosa GTPase regulator (*RPGR*) [25] (RetNet: https://sph.uth.edu/Retnet/). Of the 25 genes, mutations in the first 10 genes are responsible for adCORD, the next 13 for arCORD, and the last two for xlCORD. The associated genomic information of the 25 genes is listed in Table S1.

In our previous study on CORD, mutations were only detected in 7 of 130 (5.38%) Chinese families with CORD by using cycle sequencing of all coding exons of five genes (*CRX, GUCY2D, GUCA1A, PRPH2,* and *KCNV2*) as well as of all exons harboring reported mutations in other 17 CORD-associated genes [26], [27], [28], [29]. Of the seven families, all mutations were identified in genes responsible for adCORD but none in genes for arCORD and xlCORD. The genetic cause for most families (the remaining 123 of 130 (94.62%) Chinese families) was still unknown. In order to identify the additional cause of most CORD and to disclose further the mutation spectrum and frequency of the 25 genes, whole exome sequencing was used to screen for mutations in 47 unrelated Chinese families with CORD.

## Materials and Methods

### Patients

Forty seven probands from unrelated families with CORD were recruited from the Eye Hospital of Zhongshan Ophthalmic Center, Sun Yat-sen University. Patients with identified mutation who were included in our previous study were excluded from this one. Written informed consents were obtained from the participants or their guardians before the study, which was conforms to the tenets of the Declaration of Helsinki and follows the Guidance of Sample Collection of Human Genetic Diseases (863-plan) by the Ministry of Public Health of China. This study was approved by the Institute Review Board of the Zhongshan Ophthalmic Center. Genomic DNA was prepared from the blood leukocytes as previous described [30].

### Exome Sequencing

Exome sequencing was completed by using a commercial service from BGI Shenzhen (http://www.genomics.cn/index.php). The exome sequencing, genotype calling, and SNP calling were in the same way as the methods reported before [31]. In brief, exome capture was carried out by using a NimbleGen SeqCap EZ Exome (44 M) array. Exon-enriched DNA fragments were sequenced by the Illumina Genome Analyzer II. The average sequencing depth was set to 60-fold. SOAP aligner was used to set the sequencing reads to UCSC hg19 [32], [33]. The likelihood of possible genotypes in the target regions was calculated using SOAPsnp [34]. Variants in all the 25 genes detected by exome sequencing were selected for validation. Exome sequencing dataset of the patients with identified mutations in this study have been deposited to NIH (http://www.ncbi.nlm.nih.gov/biosample: accession number SAMN01997562 to SAMN01997571).

### Sanger Sequencing

Sanger sequencing was used to validate variants in the 25 genes that resulted from exome sequencing, including heterozygous variants in the adCORD genes, homozygous or compound heterozygous variants in the arCORD genes, or hemizygous variants in the xlCORD genes. Primers (Table S2) used to amplify the regions containing the variants were designed by primer design tool Primer3 (http://frodo.wi.mit.edu/primer3/) [35]. A touch-down polymerase chain reaction (PCR) was used to amplify the fragments with variants, as previously reported [36], and the amplicons were analyzed with an ABI BigDye Terminator cycle sequencing kit v3.1 (Applied Biosystems, Foster City, CA) on an ABI3100 Genetic Analyzer (Applied Biosystems). Sequencing results from patients and controls were compared using the SeqManII program of the Lasergene package (DNAStar Inc, Madison, WI). Detected variants were further sequenced in the available family members. Novel variants were further evaluated in 192 control individuals. The description of mutations was in accordance with the nomenclature for the description of sequence variants [37](HGVS: http://www.hgvs.org/mutnomen/). The conservation of a variation was evaluated by Phastcons_score (http://varianttools.sourceforge.net/Annotation/PhastCons) [38], the effect of a missense variation was analyzed by using SIFT [39] (http://sift.jcvi.org/) and Polyphen-2 [40] (http://genetics.bwh.harvard.edu/pph2/) online tools, and the effect of splicing site changes was predicted by Berkeley Drosophila Genome Project (BDGP) [41] (http://www.fruitfly.org/).

Considering that CORD-causing mutations are rare and the presence of the normal carriers of arCORD gene mutations, we assumed that the affected individuals were likely homozygous or compound heterozygous, so variants absent in the dbSNP134, 1000Genome or with allelic frequencies ≤0.006 were considered to be potentially pathogenic (frequency of heterozygote carriers calculated based on a disease incidence of 1∶40,000, under the hypothesis that a unique arCORD gene would explain the remaining 40% of cases [42]).

## Results

**Figure 1:**
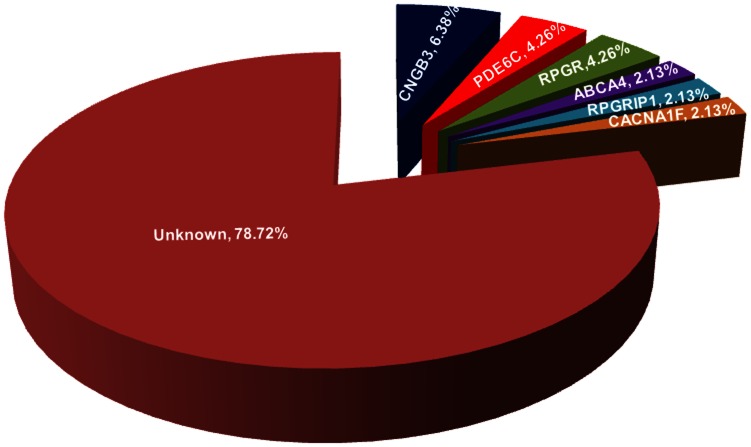
Prevalence of mutations in the investigated genes in our cohort of 47 CORD patients.

**Figure 2:**
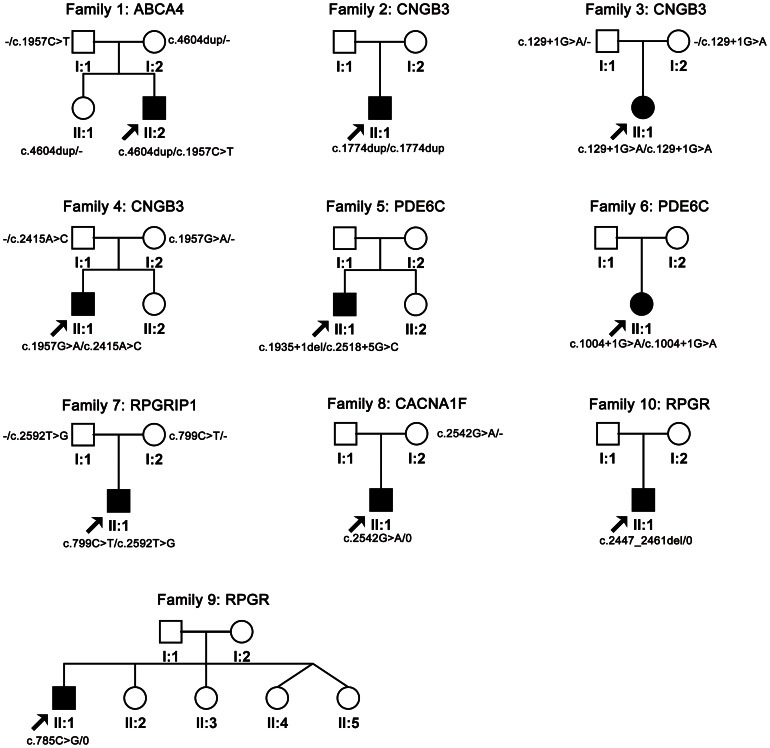
Pedigrees of the 10 families with mutations. The family numbers and their corresponding mutations were shown just above the pedigrees.

Whole exome sequencing identified 14 potential pathogenic mutations in 10 of the 47 (21.28%) families with CORD (Table 1), including seven homozygous or compound heterozygous mutations in four (*ABCA4, CNGB3, PDE6C,* and *RPGRIP1*) of the 13 genes associated with arCORD, and three hemizygous mutations in the two genes (*RPGR* and *CACNA1F*) associated with xlCORD. Of the 14 mutations, nine were novel. The 14 mutations in the 10 families involved six of the 25 CORD-associated genes, including *CNGB3* (three families), *PDE4C* (two families), *RPGR* (two families), *ABCA4* (one family), *RPGRIP1* (one family), and *CACNA1F* (one family) (Figure 1), respectively. Sanger sequencing confirmed the 14 mutations in the 10 families (Figure S1). Segregation analysis was available for five of the 10 families in where the mutations co-segregated with the disease in the family (Figure 2). No potential pathogenic mutation was identified in the other 19 genes of the 47 families.

**Table 1:** Potential pathogenic mutations detected in 10 of the 47 families.

Family ID	Gene	Variations		Status	Bioinformation analysis				Allele frequency in		Reference
		DNA	Protein		SIFT	Polyphen-2	Splice	Phastcons _score	patients	controls	
Family 1	ABCA4	c.4604dup	p.T1537Nfs*18	hetero	–	–	–	0.997	1/94	0/384	novel
Family 1	ABCA4	c.1957C>T	p.R653C	hetero	D	PD	–	1.000	1/94	NA	[38]
Family 2	CNGB3	c.1774dup	p.E592Gfs*44	homo	–	–	–	1.000	2/94	0/384	novel
Family 3	CNGB3	c.129+1G>A	–	homo	–	–	DSA	1.000	2/94	0/384	novel
Family 4	CNGB3	c.2415A>C	p.E805D	hetero	D	PD	–	1.000	1/94	NA	rs186448979#
Family 4	CNGB3	c.1957G>A	p.A653T	hetero	tolerated	benign	–	0.000	1/94	0/384	novel
Family 5	PDE6C	c.1935+1del	–	hetero	–	–	DSA	1.000	1/94	0/384	novel
Family 5	PDE6C	c.2518+5G>C	NA	hetero	–	–	DSA	0.112	1/94	0/384	novel
Family 6	PDE6C	c.1004+1G>A	–	homo	–	–	DSA	1.000	2/94	0/384	novel
Family 7	RPGRIP1	c.2592T>G	p.Y864*	hetero	–	–	–	0.994	1/94	0/384	novel
Family 7	RPGRIP1	c.799C>T	p.R267*	hetero	–	–	–	1.000	1/94	NA	[36]
Family 8	CACNA1F	c.2542G>A	p.G848S	hemi	tolerated	benign	–	1.000	1/94	0/384	novel
Family 9	RPGR	c.785C>G	p.A262G	hemi	tolerated	benign	–	0.002	1/94	NA	[39]
Family10	RPGR	c.2447_2461del	p.G816_E820del	hemi	–	–	–	NA	1/94	NA	[40]

Note: D = damaging; PD = probably damaging; DSA = donor site abolished.

# The variation was found in 1000 Genomes database with the Global minor allele frequency (MAF) of G = 0.001/3 so that the pathogeneity of the variants in this family need to be clarified further.

Clinical data of the 10 probands with potential pathogenic mutations are listed in Table 2. All probands with identified mutations had an early onset severe form of retinal dystrophy with predominantly cone involvement. Fundus changes were mainly in the macular regions, showing mild pigmentary changes and a loss of foveal reflex, as well as attenuated retinal arteries in rare cases.

**Table 2:** Clinical data of the probands with CORD and identified potential pathogenic mutations.

Family ID	Gene	Nucleotide changes	Sex	Age (year) at		First	Best visual acuity		Fundus changes		ERG responses from	
				exam	onset	symptom	right	left	right	left	rod	cone
Family 1	ABCA4	c.[1957C>T];[4604dup]	M	7.0	6.0	PV, PP	0.20	0.15	MA, TPOD, ARA	MA, TPOD, ARA	Normal	Reduced
Family 2	CNGB3	c.[1774dup];[1774dup]	M	6.5	EC	PV,NYS	0.20	0.20	MA, TPOD	MA, TPOD	Mildly reduced	Extinguished
Family 3	CNGB3	c.[129+1G>A];[129+1G>A]	F	5.0	0.3	PV,NYS, PP	0.20	0.20	MA, TPOD, ARA	MA, TPOD, ARA	Normal	Extinguished
Family 4	CNGB3	c.[1957G>A];[2415A>C]	F	4.5	0.5	PP,NYS	0.10	0.20	ARA	ARA	Mildly reduced	Severely reduced
Family 5	PDE6C	c.[1935+1del];[2518+5G>C]	M	7.0	EC	NYS	0.05	0.05	High myopic	High myopic	Normal	Severely reduced
Family 6	PDE6C	c.[1004+1G>A];[1004+1G>A]	F	2.0	EC	PV,PP,NYS	PO	PO	NA	NA	Mildly reduced	Extinguished
Family 7	RPGRIP1	c.[799C>T];[2592T>G]	M	3.6	FMB	PV, PP, NYS	NA	NA	ARA,CRD	ARA,CRD	Extinguished	Extinguished
Family 8	CACNA1F	c.[2542G>A]; [0]	M	2.3	NA	PP, NYS	NA	NA	MA	MA	Severely reduced*	Extinguished
Family 9	RPGR	c.[785C>G]; [0]	M	28.0	EC	PV, PP	0.10	0.10	MA, TPOD, ARA	MA, TPOD, ARA	Moderately reduced	Extinguished
Family 10	RPGR	c.[2447_2461del]; [0]	M	9.0	EC	PV, PP	0.20	0.20	MA	MA	Normal	Extinguished

Note: F = female; M = male; EC = early childhood; FMB = first few months after birth; PV = poor vision; PP = photophobia; NYS = nystagmus; PO = pursuing object; NA = not available; MA = macular atrophy; TPOD = temporal pallor of optic disc; ARA = attenuated retinal arteries; CRD = carpet-like retinal degeneration.

* This patient did not have the “electronegative” ERG in the standard combined response.

## Discussion

Based on an initial screening of exome sequencing and the subsequent confirmation of Sanger sequencing, potential pathogenic mutations were identified in 10 of the 47 (21.28%) families with CORD, involving 14 mutations in six of the 25 CORD-associated genes. For the 47 families in this study, the contributions of causative mutations in individual genes are as follows: *CNGB3* (6.38%), *PDE6C* (4.26%), *RPGR* (4.26%), *ABCA4* (2.13%), *RPGRIP1* (2.13%), and *CACNA1F* (2.13%).

The frequency of mutations detected in this study is significantly higher than in our previous study, in which mutations were only detected in seven of 130 (5.38%) Chinese families with CORD through the process of sequencing the coding exons of five genes and all exons with reported mutations in 17 other genes [26], [27], [28], [29]. In addition, in this study, mutations in seven of the 10 families were found in arCORD-associated genes but no mutations were detected in the adCORD-associated genes; in contrast, mutations in all seven families from our previous study were found in adCORD-associated genes. This difference may partly have resulted from 1) several genes associated with adCORD having been systemically analyzed before and all patients with mutations in these genes being excluded from this study; and 2) selective analysis of exons with reported mutations perhaps failing to identify both mutations in arCORD associated genes if one mutation in the exons is not analyzed. The real proportion of mutations in the 25 genes should be higher than 21.28% in Chinese patients with CORD if mutations identified in previous study are taken into account [26], [27], [28], [29]. Further analysis of the results from exome sequencing for those patients without identified mutations in the 25 genes may provide useful clues in the identification of new genes responsible for CORD.

Previously studies on an individual gene or a set of genes have revealed a different frequency of mutation in CORD-associated genes in different populations. Sanger sequencing of 10 adCORD-associated genes identified mutations in 25 of 52 (48%) German families with adCORD, and mutations were found in *GUCY2D* (24%), *PRPH2* (12%), *GUCA1A* (8%), *CRX* (4%), and *PROM1* (2%) [43]. In other studies for individual genes, mutations in *CRX* are responsible for a 4.76% proportion of CORD [26], *GUCY2D* for 11.11% of CORD in a Japanese population [44] and 9.09% in a Spanish one [45], *GUCA1A* for 16.67% of CORD in a German one [46], *PRPH2* for 11% of CORD in another German population [43], *AIPL1* for 1.82% of CORD in an American sample [1], *PROM1 for* 0.93% of CORD in a Dutch population [16], *SEMA4A* for 8.00% of CORD in a Pakistani group [9], and *UNC119 for* 5.00% of CORD in another American population [10].

For genes known to be associated with arCORD, their mutations might be responsible for nearly 40% of arCORD [42], whereas mutations in ABCA4 are most frequent in European and American populations, ranging from 16.13% to 65.00% [47], [48], [49], [50]. However, ABCA4 mutations are very rare in Chinese families with CORD. The mutation frequency of CNGB3 [49], *PDE6C* [20], [49], and *PRGRIP1* [23] in other populations are relatively rare, as seen in Chinese sample. For other CORD-associated genes without identified mutations in this study, mutations in other populations are also rare, as with *RAX2* in 0.62% of CORD [21], *CERKL* in 1.85% of Canadian CORD [16], or *ADAM9* [12], *RDH5* [22], *CDHR1* [15], and *C8ORF37* [13] in a few reports based on an analysis of a limited number of families.

## Supporting Information

Figure S1 **Sequence chromatography.** Forteen sequence changes detected in the probands with CORD are shown (left column) compared with corresponding normal sequences (right column). Some known mutations were not verified in the normal controls, so the normal sequences are absent.
(TIF)

Table S1 **Genomic information of the 25 genes referred in this study.** This table listed the accession numbers of the genomic DNA, mRNA, and protein for each of the 25 genes. The information is based on human genome reference GRCh37.p10.
(XLS)

Table S2 **Sequences of primers used in this study.** This table listed 52 primers used to amplify the genomic fragments with variants detected by exome sequencing.
(XLS)
