# Supplementary figures and images for: Exome Sequencing of 47 Chinese Families with Cone-Rod Dystrophy: Mutations in 25 Known Causative Genes
Source: PLoS One. 2013 Jun 11;8(6):e65546. doi: 10.1371/journal.pone.0065546 (PMC3679152; doi:10.1371/journal.pone.0065546)

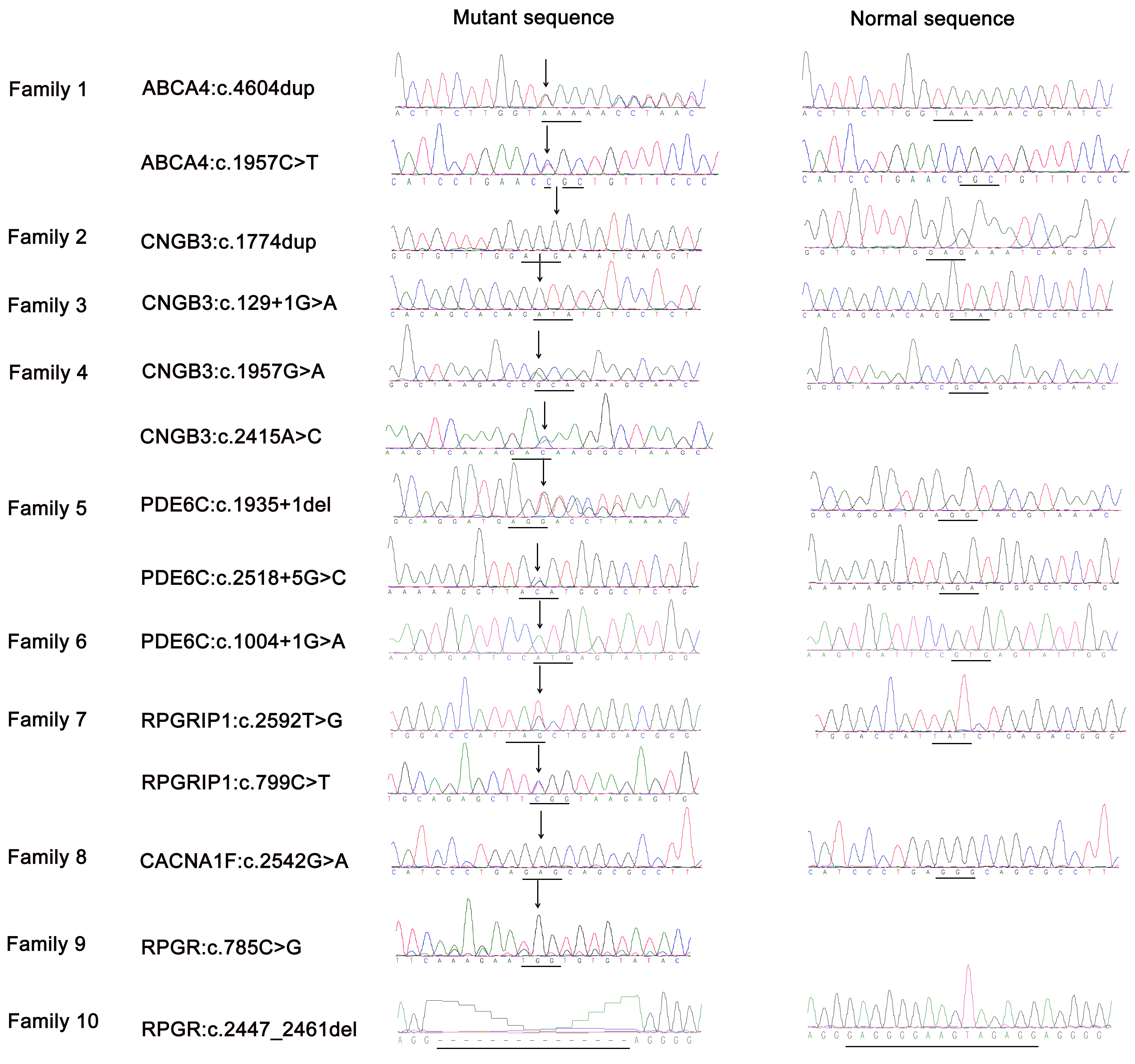

Supplement: Figure S1 — Sequence chromatography. Forteen sequence changes detected in the probands with CORD are shown (left column) compared with corresponding normal sequences (right column). Some known mutations were not verified in the normal controls, so the normal sequences are absent. (TIF) [file pone.0065546.s001.tif]
